# Hyaluronidases improve the hyaluronic acid yield during the fermentation of *Streptococcus zooepidemicus*

**DOI:** 10.3389/fbioe.2025.1625009

**Published:** 2025-07-16

**Authors:** Chuan-Li Kang, De-Qiang Zheng, Zhi-Yuan Yao, Kang Yang, Yuxue Zhao, Zihan Mao, Yang Liu, Haijun Li, Jin-Song Gong, Lei Liu, Qingwen Jia, Zheng-Hong Xu, Jin-Song Shi, Le Xue

**Affiliations:** ^1^ Key Laboratory of Carbohydrate Chemistry and Biotechnology of Ministry of Education, School of Life Sciences and Health Engineering, Jiangnan University, Wuxi, China; ^2^ Shandong Freda Pharmaceutical Co., Ltd., Jinan, China; ^3^ Shandong Focusfreda Biotech Co., Ltd, Qufu, China; ^4^ School of Biotechnology, Jiangnan University, Wuxi, China; ^5^ College of Biomass Science and Engineering, Sichuan University, Chengdu, China

**Keywords:** hyaluronidases, hyaluronic acid, enzymatic fermentation, protein expression, fermentation process optimization

## Abstract

**Background:**

Hyaluronic acid (HA), a linear acidic mucopolysaccharide with exceptional biocompatibility, is extensively utilized in pharmaceuticals and cosmetics. Industrial HA production predominantly relies on Streptococcus zooepidemicus fermentation. However, the accumulation of high-molecular-weight (HMW) HA increases broth viscosity, impeding nutrient diffusion and limiting yield.

**Methods:**

To address this, four HAases, HHya1, LHya2, SHya3, and EHya4, were expressed and screened for enzymatic activity. we evaluated the strategic addition of hyaluronidases (HAases) to degrade HMW HA during fermentation, thereby reducing viscosity and enhancing productivity.

**Results:**

HHya1 and EHya4 exhibited superior expression levels and catalytic efficiency. Purification and functional characterization revealed distinct degradation profiles, HHya1 hydrolyzed HMW HA into saturated tetrasaccharides, while EHya4 generated unsaturated disaccharides. In shake-flask fermentations, supplementation with 1500 U/L EHya4 increased HA titer by 12%, outperforming HHya1. Scaling to bioreactor cultivation with viscosity-controlled HAase dosing further optimized productivity. By administering HAase at intervals corresponding to viscosity thresholds, HA titers reached 10.3 g/L, representing a 14.4% increase over baseline.

**Conclusion:**

These findings demonstrate that HAase application alleviates viscosity-associated bottlenecks in S. zooepidemicus fermentations, establishing an optimized process for scalable HA production. This approach balances enzymatic degradation with microbial growth kinetics, offering a practical strategy for industrial HA biosynthesis.

## 1 Introduction

Hyaluronic acid (HA) is a naturally occurring linear polymer comprising repeating disaccharide units of D-glucuronic acid (GlcUA) and N-acetyl-D-glucosamine (GlcNAc) linked by β-1,4 and β-1,3 bonds (Meyer, 1954). Known for its exceptional viscoelasticity, potent moisturizing properties, and biocompatibility, HA finds wide application in various sectors such as medicine, cosmetics, and nutritional health products (Long Liu et al., 2011). Its versatile utility extends to ophthalmology, joint disorders, skin rejuvenation, vascular prosthetics, adipose tissue regeneration, nerve reconstruction, and cancer therapy (Abatangelo et al., 2020). HA fragments are leveraged to address wrinkles, expression lines, fibroblast depletion, and scars, and they hold significant commercial value (Yasin et al., 2022). The biological attributes of HA are intricately linked to its chain length and molecular weight, with distinct functions exhibited across varying molecular weight ranges (Qiu et al., 2021). High molecular weight HA (HMW-HA, MW ≥ 1 × 10^6^ Da) showcases remarkable viscoelasticity, hydration, anti-inflammatory properties, and lubrication, making it ideal for intra-articular injections to restore joint tissue viscoelasticity and repair cartilage degeneration. HMW-HA also serves in cosmetic and dermatological applications by promoting wound healing, postoperative anti-adhesion, and sustained drug release (Stern et al., 2006). In contrast, low molecular weight HA (LMW-HA) with molecular weights between 1 × 10^4^ and 1 × 10^6^ Da exhibits enhanced bioavailability and plays a pivotal role in chronic wound healing and the development of HA crosslinking agents (Buffa et al., 2019; Ke et al., 2013; Yasin et al., 2022; Zhang et al., 2025). LMW-HA holds significant practical value. Hyaluronidases facilitate the conversion of HMW-HA into LMW-HA (El-Safory et al., 2010).

Hyaluronidases, a diverse group of glycoside hydrolases, degrade glycosidic bonds within HA polymers to generate HA of varying molecular weights (El-Safory et al., 2010). Their role in correcting cosmetic fillers, aiding in medicine diffusion and absorption, and reducing postoperative pain in the pharmaceutical cosmetic industry underscores their practical significance (Cavallini et al., 2013). Historically, hyaluronidases for medical use were initially sourced from crude extracts of ovine or bovine testicular tissue (Cavallini et al., 2013). The enzymatic action of hyaluronidases primarily involves degrading HA through cleaving β-1,4 glycosidic bonds or β-1,3 glycosidic bonds (El-Safory et al., 2010; Kang et al., 2016). Hyaluronidases are classified into three categories based on their substrate specificity, catalytic mechanisms, and the types of degradation products they produce. The first category of hyaluronidases consists of mammalian hyaluronidases, which degrade HA by cleaving β-1,4-glycosidic bonds, with the primary products being tetrasaccharide molecules (El-Safory et al., 2010). The second category includes hyaluronidases found in leeches and the salivary glands of hookworms, which degrade HA by cleaving β-1,3-glycosidic bonds, with the main products being tetrasaccharides and hexasaccharides (da Silveira et al., 2007; Ferrer et al., 2013; Kang et al., 2016; KREIL, 1993). Microbial-derived hyaluronidases, the third category, degrade HA through a β-elimination reaction, yielding unsaturated disaccharides (El-Safory et al., 2010; Kumon et al., 2024). Hyaluronidase’s wide-ranging applications span medicine, cosmetology, and the production and preparation of HA with diverse molecular weights (Jung, 2022). Recent studies have explored hyaluronidase’s role in optimizing HA titer (Wang et al., 2020).

Industrial HA production predominantly relies on microbial fermentation due to the rising demand across pharmaceutical, medical, food, and cosmetic industries, necessitating enhanced production efficiency (Fallacara et al., 2018). Various strains are employed in fermentative HA production, with *S. zooepidemicus*, *Bacillus subtilis*, and *Corynebacterium glutamicum* being prominent choices for industrial-scale production (Jin et al., 2016; Serra et al., 2023). Among them, *S*. *zooepidemicus* has the advantages of short production period and high yield, so use it product HA is the mainstream way for industrial production of HA at present (Sugahara et al., 1979; Sze et al., 2016). During *S*. *zooepidemicus* fermentation of HA, elevated HA concentrations increase broth viscosity, substantially reducing DO levels and oxygen mass transfer efficiency. It impairs bacterial metabolism and hinders HA accumulation. DO concentration and oxygen transfer coefficients critically regulate intracellular redox potential and energy charge, governing metabolic activity. Under adequate oxygen, *Streptococcus* cells aggregate, promoting extensive HA encapsulation as a protective layer; simultaneously, severe oxygen limitation induces anaerobic respiration and by-product formation. Consequently, viscosity-driven DO depletion and impaired oxygen transfer constitute a major bottleneck for microbial HA production. (Chong et al., 2005; Wang et al., 2020; Zhang et al., 2023). To optimize production cost-effectively, strategies encompass selecting production strains, refining culture conditions, purification processes, and supplementing with additional enzymes to boost HA yield (Serra et al., 2023; Yao et al., 2021). For instance, incorporating hyaluronidase and refining S. zooepidemicus fermentation through a two-stage semi-continuous approach significantly enhances HA yield (Zhang et al., 2023). Although the addition of hyaluronidase can increase the yield of HA, research on this area is limited and thus warrants further development.

To address these bottlenecks, this study pioneered the expression and application of novel HAases from *Hirudo nipponia*, venomous spiders (*Loxosceles intermedia*), *Synanceia horrida*, *Enterobacterales*, and honeybees. Through optimized expression systems and viscosity-controlled enzymatic dosing in bioreactors, HAase supplementation reduced broth viscosity by 53%, improved mass transfer efficiency, and enabled concurrent production of high-titer HA (10.3 g/L, 14.4% yield increase) with a narrow LMW-HA distribution (1 × 10^4^–2 × 10^5^ Da). This dual strategy of enzymatic viscosity management and targeted depolymerization establishes a scalable platform for industrially producing HA with controlled MW, reconciling yield optimization with product specificity for diverse biomedical applications.

## 2 Results and discussion

### 2.1 Screening and expression of different hyaluronidases

**FIGURE 1:**
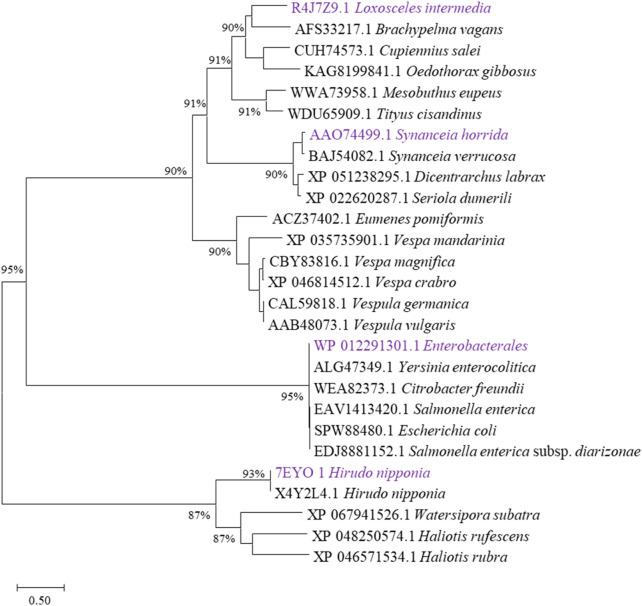
Evolutionary tree analysis. Selection of different types of hyaluronidases that have been characterized functionally, sequences were downloaded in National Center for Biotechnology Information (NCBI, https://www.ncbi.nlm.nih.gov/). The ones labeled purple are the hyaluronidases selected in this study.

**FIGURE 2:**
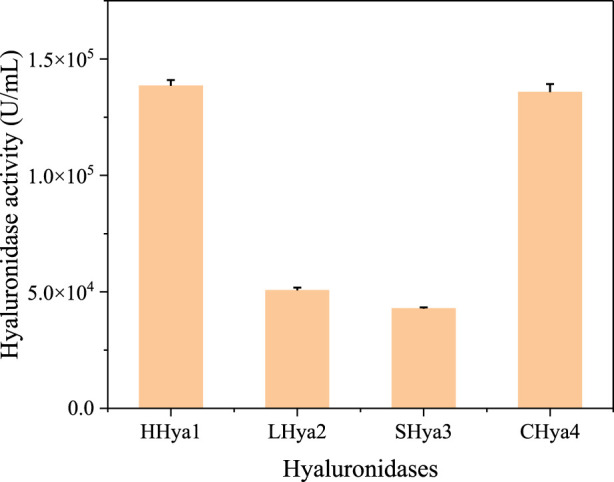
Crude enzyme activity of hyaluronidase from different sources. Among them, HHya1, LHya2 and Shya3 were selected for enzyme activity assay at the time of highest enzyme expression, respectively. EHya4 was the cell lysate after 16 h of culture.

Enzymatic degradation shows significant advantages in biological environments. It can achieve rapid decomposition of pollutants under mild conditions, significantly reducing energy consumption and treatment costs (Han et al., 2021; Payanthoth et al., 2024). Combined with microbial fermentation, it provides an efficient, precise, environmentally friendly and sustainable pollution control and material recycling solution (Xia et al., 2017). Numerous studies have investigated hyaluronidase, and in this study, we analyzed evolutionarily validated hyaluronidases through phylogenetic clustering (Figure 1) and selected four functionally characterized candidates for heterologous expression: HHya1 (EC 3.2.1.36) from *H. nipponia* (Jin et al., 2014), LHya2 from *L. intermedia* (Ferrer et al., 2013), SHya3 from *S. horrida* (Ng et al., 2005), and EHya4 from *Enterobacterales*. HHya1, identified as hyaluronate-3-glycanohydrolases (EC 3.2.1.36), acts as an endo-β-D-glucuronidase primarily degrading HA into tetrasaccharides (Jin et al., 2014; Wenshuang Wang and Li, 2017). The HHya1 can express in *P. pastoris*. The LHya2 is present in spider venom and has been shown to degrade hyaluronic acid to produce a small molecule of 29–45 kDa (Ferrer et al., 2013). SHya3, discovered in the venom gland of stonefish Synanceja horrida, marks the first hyaluronidase from an aquatic source, capable of breaking down HA less than 20 kDa into smaller oligosaccharides (Ng et al., 2005). HHya1, LHya2 and SHya3 are derived from eukaryotes. While HHya1 has shown good activity when expressed using *Pichia pastoris* (Kang et al., 2016). LHya2 and SHya3 have been successfully expressed in *Escherichia coli*, yielding inclusion bodies that are subsequently denatured, refolded *in vitro*, and transformed into active hyaluronidase, although with modest enzyme activity (da Silveira et al., 2007; Ng et al., 2005). To address this, we engineered LHya2 and SHya3 with sequence truncations and SUMO fusion tags to enhance solubility in *P. pastoris*, though activity improvements remained limited. In contrast, EHya4—a prokaryotic enzyme—achieved robust expression in *E. coli* without optimization. To determine the expression level of the recombinant proteins, the crude lysates supernatant activity of the four recombinant proteins was determined. Comparative analysis revealed superior expression levels and activities for HHya1 (138,467 U/mL) and EHya4 (135,733 U/mL) versus LHya2 (51,982 U/mL)/SHya3 (42,935 U/mL) (Figure 2; Supplementary Figure S1). Given industrial cost considerations, HHya1 and EHya4 were selected for subsequent *S. zooepidemicus* fermentation trials, balancing enzymatic efficiency with production feasibility for HA yield optimization.

### 2.2 Hyaluronidase production and purification process

**FIGURE 3:**
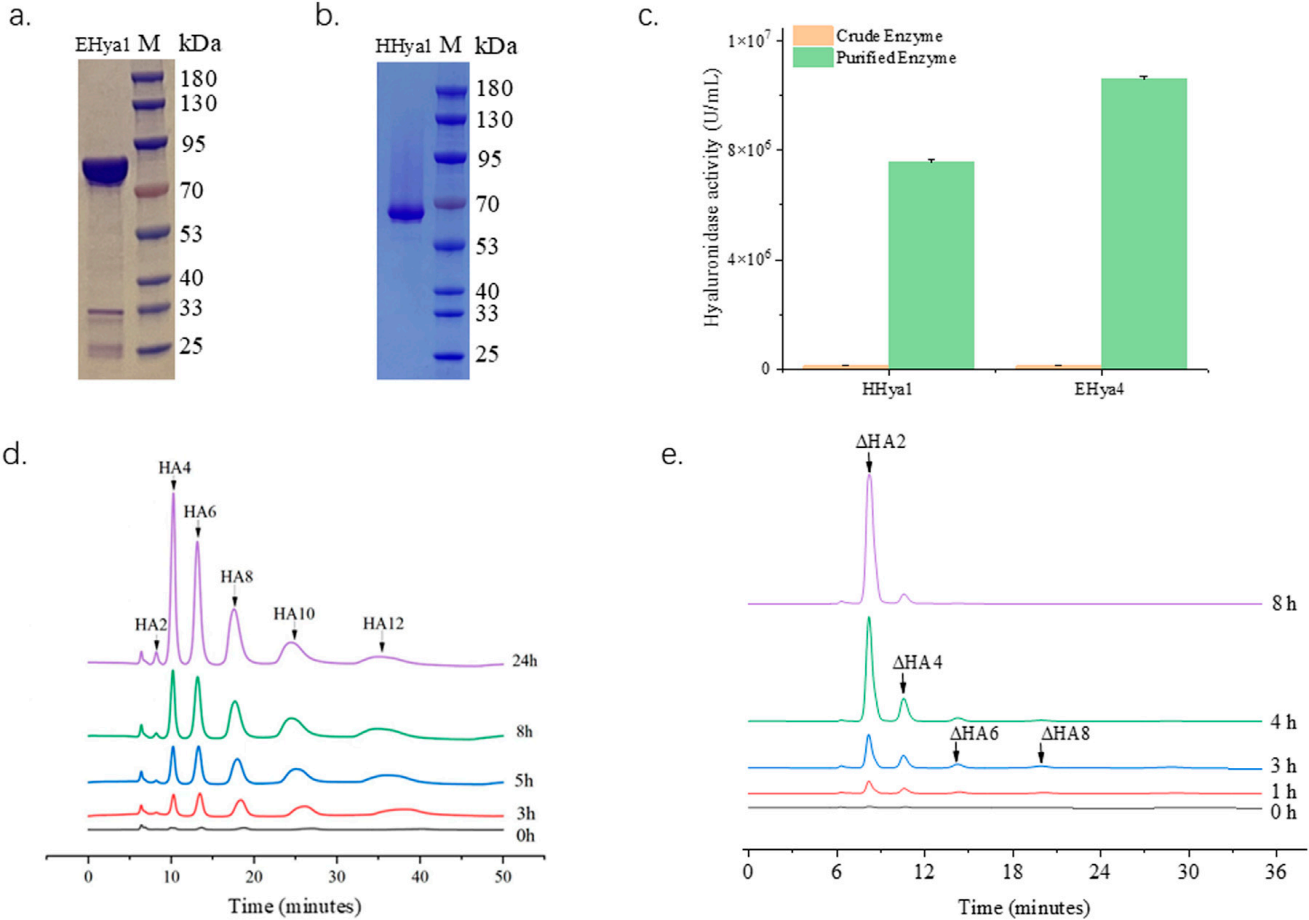
Purification of hyaluronidase HHya1 and EHya4 and enzyme reaction products. Purified SDS-PAGE electropherograms of HHya1 **(a)** and EHya4 **(b)**. Comparison of enzyme activity of crude enzyme solution and purified enzyme solution **(c)**. The detection peak plots of HHya1 hydrolyzed HA at different times **(d)**. The detection peak plots of EHya4 hydrolyzed HA at different times **(e)**.

Hyaluronic acid fermentation optimization requires precise control of enzyme additives to maintain system stability. Therefore, it is necessary to obtain highly active enzymes with the minimum addition amount. In this study, the fermentation process was optimized by adding purified enzymes with the same enzyme activity. In this study, evolutionary analysis of hyaluronidases (Figure 1) identified four candidates—HHya1 (*H. nipponia*), LHya2 (*L. intermedia*), SHya3 (*S. horrida*), and EHya4 (Enterobacterales)—for heterologous expression with N-terminal His-tags to facilitate purification. Ni-NTA affinity chromatography significantly enhanced enzyme purity (Figures 3a,b), boosting specific activity by 54-fold for HHya1 (138,467 U/mL) and 77-fold for EHya4 (135,733 U/mL) compared to crude extracts (Figure 3c). Functional characterization revealed distinct degradation profiles: HHya1 generated saturated HA oligosaccharides (Figure 3d), while EHya4 predominantly produced unsaturated disaccharides (ΔHA2) within 8 h, with minor tetrasaccharide (ΔHA4), hexasaccharide (ΔHA6), and octasaccharide (ΔHA8) byproducts (Figure 3e). Despite lower activities of LHya2 (51,982 U/mL) and SHya3 (42,935 U/mL), their inclusion highlighted mechanistic diversity in HA depolymerization. In this study, the kinetic parameters of four different hyaluronidases were determined (Table 1).

**TABLE 1:** Kinetic parameters of different enzymes.

Enzymes	Km (μM)	Vmax (μM·min^−1^)	Kcat (s^−1^)	kcat/Km (mL·mg^−1^s^−1^)
HHya1	2.10	3.26	6.52	6.21
LHya2	6.35	1.39	2.78	0.87
SHya3	12.88	0.71	1.41	1.41
EHya4	1.26	2.87	5.74	5.74

In *Streptococcus zooepidemicus* fermentations, purified HHya1 and EHya4 reduced broth viscosity by 53%, improved mass transfer efficiency, and achieved 10.3 g/L HA titers with a narrow molecular weight distribution (1 × 10^4^–2 × 10^5^ Da). While this study focused on yield optimization, the enzymatic specificity of HHya1 (saturated products) and EHya4 (unsaturated ΔHA2) positions them as versatile tools for tailored HA production in biomedical and cosmetic applications. Ongoing process refinements aim to standardize molecular weight control, underscoring the potential of enzymatic engineering to harmonize industrial scalability with product customization in HA biosynthesis.

### 2.3 EHya4 is more beneficial for HA production

**FIGURE 4:**
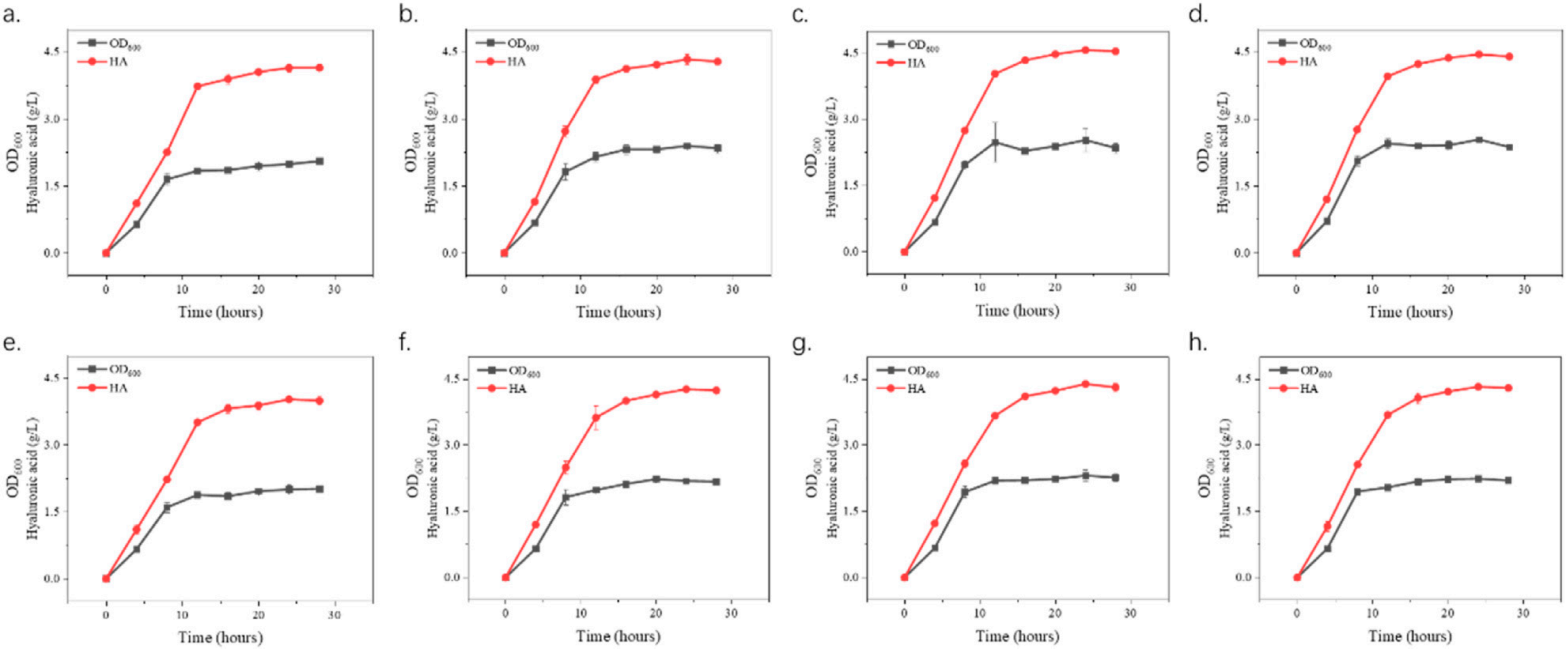
Growth curves and hyaluronic acid (HA) production. S. zooepidemicus was cultured using shake flask fermentation and different concentrations of hyaluronidase from different sources were added when the fermentation was done at 8 h and 12 h. The fermentation was carried out in the same way as the fermentation. In **(a–d)**, EHya4 was added at 500 U/L, 1,000 U/L, 1,500 U/L, and 2,500 U/L, respectively. **(e–h)**, HHya1 was added at 500 U/L, 1,000 U/L, 1,500 U/L, and 2,500 U/L, respectively.

**FIGURE 5:**
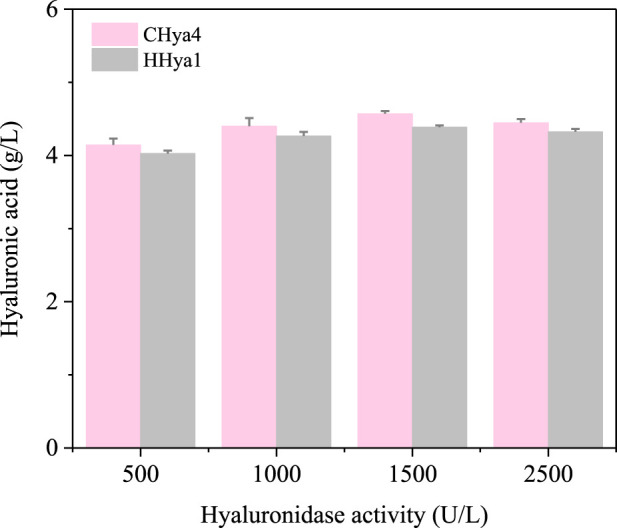
Maximum yield of hyaluronic acid (HA) at 24 h with the addition of different concentrations of HA. The pink color is the yield of HA when EHya4 was added, and the gray color is the yield of HA when HHya1 was added.

The optimization of fermentation conditions, as the core bridge connecting laboratory scale and industrial large-scale production, determines the economic feasibility of the fermentation process, the stability of product quality and the key links of the entire biomanufacturing process (Kwaw et al., 2017; Wang et al., 2023). To assess the role of hyaluronidases in enhancing HA production during *S*. *zooepidemicus* fermentation, shake flask experiments were conducted with varying concentrations of HHya1 and EHya4. The different catalytic efficiency represented by the differences in kinetic parameters of different enzymes leads to differences in viscosity and DO levels during fermentation, which leads to differences in HA titer. It was found that the maximum HA production was categorized as 4.03, 4.27, 4.39, and 4.32 g/L when 500, 1,000, 1,500, and 2,500 U/L of HHya1 were added, respectively (Figures 4a–d). Among them, the highest yield of HA was obtained when 1,500 U/L was added, which increased the yield by 8% compared to the fermentation process without HA (Figure 5). On the other hand, the maximum yield of HA was categorized as 4.14, 4.33, 4.57, and 4.45 g/L when 500, 1,000, 1,500, and 2,500 U/L of EHya4 were added, respectively (Figures 4e–h). As with HHya1, the highest yield of HA was achieved when EHya4 was added at 1,500 U, with a 12% increase in yield compared to the fermentation process without HA (Figure 5). It was found that the highest hyaluronan production was achieved when the addition of both hyaluronans was controlled at 1,500 U/L, so hyaluronidase needs to be added moderately (Figure 4). Therefore, EHya4 can be used as the optimal enzyme to enhance the HA production capacity of *S. zooepidemicus* by adding hyaluronidase. The enzyme was used as the main ingredient to optimize the HA production process. Notably, hyaluronidase supplementation correlated with elevated biomass (OD600), suggesting enzymatic HA degradation alleviates broth viscosity, enhances nutrient diffusion, or releases growth-promoting oligosaccharides, thereby partially addressing the chronic challenge of low cell density in *S. zooepidemicus* fermentations. These findings reveal the dual role of hyaluronidases in boosting HA yield and microbial vitality, though the pathogenicity and metabolic constraints of the strain necessitate further exploration of novel enzymes or engineered systems. Future studies should prioritize hyaluronidases with enhanced thermostability or synergistic activity to amplify fermentation density and HA output, ultimately advancing scalable, cost-effective bioproduction of tailored HA for biomedical and cosmetic applications.

### 2.4 Scale-up process for increasing HA production by addition of hyaluronidase

**FIGURE 6:**
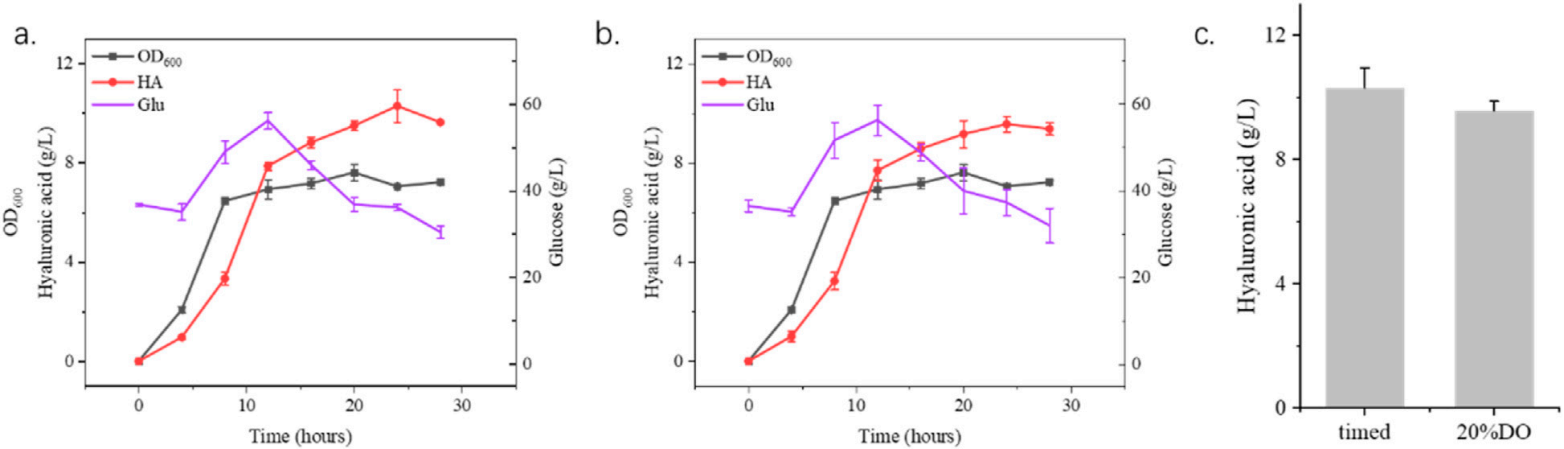
Fermentation process optimization using 15 L fermenter. **(a)** shows the timed addition of hyaluronidase with 1,500 U/L every 4 h from the 8th hour until 24 h. **(b)** shows the addition of hyaluronic acid according to the amount of dissolved oxygen with 1,500 U/L hyaluronidase when the dissolved oxygen was 20%. **(c)** shows the highest yield of hyaluronidase in two different process ways.

Building on shake flask experiments demonstrating the benefits of moderate hyaluronidase supplementation for hyaluronic acid (HA) production in *S. zooepidemicus*, this study systematically evaluated enzymatic strategies in a 15 L fermenter. Two fermentation strategies, timed addition (fixed intervals) and dissolved oxygen (DO)-responsive addition, were compared to optimize HA yield. The timed addition of 1,500 U/L hyaluronidase at predetermined intervals achieved a HA titer of 10.3 g/L, representing a 14.4% increase over non-supplemented fermentations. In contrast, DO-responsive addition, where enzyme dosing was triggered by dissolved oxygen thresholds, yielded 9.9 g/L HA, a 10% improvement (Figure 6). The superior performance of timed supplementation likely stems from sustained viscosity reduction and consistent nutrient availability, whereas DO-based delays in enzyme administration may allow transient HA accumulation to impede mass transfer. Notably, these results align with shake flask trends, confirming hyaluronidase’s role in mitigating viscosity-related metabolic constraints. However, this study focused solely on enzymatic supplementation, leaving other parameters (pH, feeding strategies, aeration) unoptimized. Further research integrating hyaluronidase dosing with multivariate process engineering could unlock additional yield gains. These findings underscore the industrial viability of timed enzymatic supplementation for HA bioproduction, while highlighting the need for holistic fermentation optimization to fully harness hyaluronidase’s potential in scalable systems.

## 3 Materials and methods

### 3.1 Strains, plasmids, primers and culture conditions

The strains used in this study are *P. pastoris* GS115 (purchased from Bioon), *S. zooepidemicus*, and *Enterobacteriaceae* (both preserved in our laboratory). The genes were synthesized by Genscript Biotech Corporation (Supplementary Table S1). The amino acid sequences of each part are shown in Supplementary Table S2. The medium used in this study was: Yeast Extract Peptone Dextrose (YPD) medium: yeast extract 10 g/L, peptone 20 g/L, glucose 20 g/L. Yeast Nitrogen Base (YNB) medium 13.4 g/L, biotin 4 × 10^−4^ g/L, and agar 20 g/L. Buffered Glycerol-complex (BMGY) medium: yeast extract 10 g/L, peptone 20 g/L, K_2_HPO_4_ 3 g/L, KH_2_PO_4_ 11.8 g/L, amino acid-free yeast nitrogen source YNB 3.4 g/L, ammonium sulfate 10 g/L, biotin 4 × 10^−4^ g/L, glycerol 10 g/L. Buffered Methanol-complex (BMMY) medium: yeast extract 10 g/L, peptone 20 g/L, K_2_HPO_4_ 3 g/L, KH_2_PO_4_ 11.8 g/L, YNB 3.4 g/L, ammonium sulfate 10 g/L, biotin 4 × 10^−4^ g/L, methanol 10 mL/L. BSM medium: glycerol 40 g/L, K_2_SO_4_ 18 g/L, KOH 4.13 g/L, 85%H_3_PO_4_ 26.7 mL/L, CaSO_4_·2H_2_O 0.93 g/L, MgSO_4_·7H_2_O 14.9 g/L, 4.4 mL/L PTM1 for filtration and sterilization. PTM1:CuSO_4_·5H_2_O 6 g/L,KI 0.09 g/L,MnSO4·H_2_O 3 g/L,H_3_BO_3_ 0.02 g/L,MoNa_2_O_4_·2H_2_O 0.2 g/L, CoCl_2_·6H_2_O 0.92 g/L, ZnCl_2_ 20 g/L, FeSO_4_·7H_2_O 65 g/L,biotin 0.2 g/L,H_2_SO_4_ 5.0 mL/L. Fermentation medium: peptone 16 g/L, yeast dip 2 g/L, glucose 10 g/L, monosodium glutamate 2 g/L, K_2_HPO_4_ 2 g/L, MgSO_4_·7H_2_O 0.7 g/L. Solid medium A: peptone 15 g/L, yeast maceration powder 5 g/L, glucose 5 g/L. K_2_HPO_4_ 2 g/L, MgSO_4_·7H_2_O 0.5 g/L, 15 g/L.

### 3.2 Competent cell preparation and electroporation

Cultivate *P. pastoris* GS115 on YPD agar plates at 30°C for 2 days, then pick a single colony and culture it in YPD medium at 30°C with shaking overnight. Inoculate the seed culture into YPD medium at a 1% inoculation ratio and culture with shaking until the OD_600_ reaches between 0.5 and 0.8. Pre-cool on ice and then centrifuge at 3,000 *g* to collect the yeast cells. Wash the yeast cells three times with pre-cooled 1 M sorbitol solution. Resuspend the cells with an appropriate amount of pre-cooled 1 M sorbitol solution, divide into portions, and add 1–2 μg of linearized plasmid that has been digested with *Sal*I enzyme and purified. Transform the plasmid into competent cells using electroporation (2,700 V, 200 Ω). After electroporation, spread on histidine-deficient MD plates for initial screening. Transfer the single colonies from the initial screening to YPD plates containing G418 (4 mg/mL) for secondary screening to select for *P. pastoris*/pPIC9K strains with high copy recombinant hyaluronidase genes.

### 3.3 Protein expression and purification

Pick the successfully constructed strain and inoculate it into 50 mL of YPD medium, cultivate at 30°C and 200 rpm for 24 h to prepare the seed culture. Transfer the seed culture to 50 mL of the initial expression medium BMGY at a volume ratio of 10%, to enrich the biomass, and cultivate at 30°C and 200 rpm for 24 h. Collect the biomass by centrifugation, wash with sterile water, and then transfer to 40 mL of the induction expression medium BMMY, cultivate at 30°C and 200 rpm, and add methanol (containing 1.2% (v/v) PTM1) at a volume ratio of 1% to the fermentation flask every 24 h until the induction expression reaches 96 h. Collect the supernatant, use membrane packaging for concentration, and then carry out enzyme activity assays. *Escherichia coli* is used for protein expression with LB medium, cultivated at 37°C and 220 rpm for 8 h, and then the biomass is collected using a centrifuge; resuspend the collected biomass with 5 times the volume of PB buffer and disrupt it using a homogenizer to release the intracellular enzymes, then collect the supernatant by centrifugation to remove cell debris. Slowly add 5% ammonium sulfate to the fermentation broth, mix the solution with an overhead stirrer at 300 rpm, let it stand for 30 min, and then centrifuge with a vertical centrifuge (5,000 rpm, 20 min) to remove the precipitate and retain the supernatant; then use 30% ammonium sulfate precipitation, retain the precipitate in the same manner, and redissolve with PB buffer at a 1:1 ratio to obtain the protein solution. Use a 300 K hollow fiber to clarify the fermentation broth containing hyaluronidase, and select different columns for purification based on the type of hyaluronidase. Verify the purified hyaluronidase using polyacrylamide gel electrophoresis.

### 3.4 Hyaluronidase activity assay and HA content detection

The unit of activity for hyaluronidase (U) is defined as the amount of enzyme required to release 1 μg of reducing sugar equivalent from the sugar chain of HA per hour under conditions of pH 5.5°C and 38°C. The reducing sugar is determined using the dinitrosalicylic acid (DNS) method, with the specific procedure as follows: Mix 0.8 mL (2 mg/mL) of HA (HA, 120 kDa) solution with 0.1 mL of supernatant and 0.1 mL of citrate buffer (pH = 5.5), react in a 38°C water bath for 15 min, then immediately boil for 5 min to stop the reaction, cool down, and after treatment, add 1 mL of the reaction mixture to 2 mL of DNS solution, mix well, and then boil in water for 10 min; after cooling in an ice water bath to room temperature, add 7 mL of deionized water, mix well, use analytical grade glucose for a standard curve, and measure the absorbance of the solution in the colorimetric tube at 540 nm with a UV-Vis spectrophotometer, and express the enzyme activity (U/mL) in terms of the amount of glucose.

The fermentation broth was treated with hyaluronidase and then tested for hyaluronic acid. For the detection of HA content, high-performance liquid chromatography (HPLC) is used. Hyaluronic acidase can act on the β-1,4-glycosidic bond of sodium hyaluronate, causing hydrolysis to produce N-acetylglucosamine-glucuronic acid disaccharides, and the content of N-acetylglucosamine-glucuronic acid disaccharide products is determined using HPLC. Detection is performed using an Agilent 1260 HPLC system (UV detector) with an MCI GEL CKO8EH column (8 mm × 300 mm, 5 μm); the mobile phase is 1% phosphoric acid; flow rate: 0.6 mL/min; injection volume: 20 μL; column temperature: 40°C; detection wavelength: 232 nm, with a peak time of 9.806 min. For the detection of HA content, high-performance liquid chromatography (HPLC) is used. Hyaluronic acidase can act on the β-1,4-glycosidic bond of sodium hyaluronate, causing hydrolysis to produce N-acetylglucosamine-glucuronic acid disaccharides, and the content of N-acetylglucosamine-glucuronic acid disaccharide products is determined using HPLC. Detection is performed using an Agilent 1260 HPLC system (UV detector) with an MCI GEL CKO8EH column (8 mm × 300 mm, 5 μm); the mobile phase is 1% phosphoric acid; flow rate: 0.6 mL/min; injection volume: 20 μL; column temperature: 40°C; detection wavelength: 232 nm, time: 9.806 min.

The enzymatic catalysis of hyaluronidase was assayed using sodium hyaluronate. 500 U of hyaluronidase was added to 0.1 g/mL of sodium hyaluronate solution and the reaction was carried out at 37°C. Samples were taken at 0, 1, 3, 4.5, and 8 h, and the reaction was allowed to stand at 100°C for 5 min to end the reaction, and then assayed using HPLC.

### 3.5 Fermentation experiments

Glycerol bacteria were taken and stored at −80°C and streaked, single colonies were picked and streaked in solid medium A. After overnight incubation at 37°C, they were inoculated into shake flasks containing 50 mL of fermentation medium and incubated at 37°C and 160 rpm for 14–16 h. From the 8 h of incubation, appropriate amounts of hyaluronidase were added. Samples were taken every 4 h for the determination of OD_600_ and HA content, OD_600_ was detected using a spectrophotometer, and hyaluronic acid was detected using liquid chromatography. Each experiment was repeated three times.

The addition of hyaluronidase during the fermentation process will reduce the viscosity of the fermentation broth, thus affecting the growth state of the bacteria. Therefore, the fermentation conditions need to be optimized, including stirring speed, aeration, time and concentration of sugar supplementation. Fermentation conditions were coupled with hyaluronidase addition to determine two enzyme addition schemes: the first was dissolved oxygen associated stirring, enzyme addition started when dissolved oxygen decreased to 10%, and the dissolved oxygen level was controlled by adding different hyaluronidases; the second was hyaluronidase addition according to fermentation viscosity (time dependent), hyaluronidase was added from the 8 h, and added every hour, for a total of 5 times. Samples were taken at 4 h intervals and then the hyaluronic acid content was measured using HPLC.

## 4 Conclusion

This study demonstrates a novel enzymatic strategy to enhance hyaluronic acid (HA) production in *S. zooepidemicus* fermentations through targeted hyaluronidase supplementation. Four functionally validated hyaluronidases, HHya1, LHya2, SHya3, and EHya4, which have been verified for their functions, and found that hyaluronidase EHya4 was the most effective in increasing HA yield after shaking flasks and fermentation. Moreover, the process of adding hyaluronidase at regular intervals according to the change of viscosity resulted in the highest increase in hyaluronic acid yield. Therefore, the use of adding different hyaluronidases to increase hyaluronic acid yield is a relatively new method at present, and the selection of better and more effective hyaluronidases can provide a new method for the fermentation and production process to increase the yield of hyaluronic acid.

## Data Availability

The original contributions presented in the study are included in the article/Supplementary Material, further inquiries can be directed to the corresponding authors.
