# Supplementary material for: Hyaluronidases improve the hyaluronic acid yield during the fermentation of Streptococcus zooepidemicus
Source: Front Bioeng Biotechnol. 2025 Jul 16;13:1625009. doi: 10.3389/fbioe.2025.1625009 (PMC12307326; doi:10.3389/fbioe.2025.1625009)
Supplement: Supplementary file 1 [file DataSheet1.docx]

# Supplemental Material

Table 1 Strains, Plasmids and Sources

| Strains | Plasmids | Sources |
| --- | --- | --- |
| *P. pastoris* GS115 |  | Purchased from Beina Biology |
| *E.coil* DH5α |  | Purchased from Vazyme |
| BL21 DE(3) |  | Purchased from Vazyme |
| *Streptococcus zooepidemicus* |  | Stored in the laboratory |
|  | pPIC9K-HHya1 | Synthesized by GenScript |
|  | pPIC9K-LHya2 | Synthesized by GenScript |
|  | pPIC9K-SHya3 | Synthesized by GenScript |
|  | pET28a-EHya4 | Synthesized by GenScript |

Table 2 Amino acid sequence in this paper

| Name | Sequence |
| --- | --- |
| HHya1 | MKEIAVTIDDKNVIASVSESFHGVAFDASLFSPKGLWSFVDITSPKLFKLLEGLSPGYFRVGGTFANWLFFDLDENNKWKDYWAFKDKTPETATITRRWLFRKQNNLKKETFDDLVKLTKGSKMRLLFDLNAEVRTGYEIGKKMTSTWDSSEAEKLFKYCVSKGYGDNIDWELGNEPDHTSAHNLTEKQVGEDFKALHKVLEKYPTLNKGSLVGPDVGWMGVSYVKGLADGAGDHVTAFTLHQYYFDGNTSDVSTYLDATYFKKLQQLFDKVKDVLKNSPHKDKPLWLGETSSGYNSGTKDVSDRYVSGFLTLDKLGLSAANNVKVVIRQTIYNGYYGLLDKNTLEPNPDYWLMHVHNSLVGNTVFKVDVSDPTNKARVYAQCTKTNSKHTQSRYYKGSLTIFALNVGDEDVTLKIDQYSGKKIYSYILTPEGGQLTSQKVLLNGKELKLVSDQLPELNADESKTSFTLSPKTFGFFVVSDANVEACKK |
| LHya2 | MLLVTLFLFFLQALVNGDSCGSNCEKSERPKRVFNIYWNVPTFMCHQYGLYFDEVTNFNIKHNSKDNFQGDKIAIFYDPGEFPALLPLNYGKYKIRNGGVPQEGNITIHLQRFIEYLDKTYPNRNFSGIGVIDFERWRPIFRQNWGNMKIYKNFSIDLVRKEHPFWNKKMIELEASKRFEKYARLFMEETLKLAKKTRKQADWGYYGYPYCFNMSPTNFVPDCDVTARDENNEMSWLFNNQNVLLPSVYIRRELTPDQRIGLVQGRVKEAVRISNKLKHSPKVFSYWWYVYQDETNTFLTETDVKKTFQEIVINGGDGIIIWGSSSDVNSLSKCTRLREYLLTVLGPIAVNVTEAVN |
| SHya3 | MLPWTDPPLHPGHPFLFTWNAPTELCGIRFGMPLDLSYFDFVSSTLKSATNQSISIFYTDRFGVFPYVNEKTGKMYNGGLPQLIDLEQHHELAEDDIEYYIPFNQLGLAVLDFEEWRPQWIRNWGSKDIYRQYSIETVLKKNSSLSKEEAAGQAKMAFERAAKKYFLRSIRIGKRLRPNRLWGYYLYPECYNYEYKKDMAGYTGECPAIEKDRNNELLWLWRESTALFPSIYLELLLRDTQQARQYVRHRIQESIRVSKLPNSAYSIPIHAYVRPVYKDSTDNYMSEFDLVNTIGEAAALGAASVVCWGDMSVVATEDSCFDARRHLEKVMNPYIMNVSTATQLCSKALCQDQGRCVRKHWDDDVFLHLDPRRYRIEQQRGGGPLTVTGDLSQDDVNWFDRNFDCMCYSEKPCRSALTFNVINKAVINKAPPACDGTARSDVHCDVNINF |
| EHya4 | MGSVHAQIATTIGHIHRISRRIGLVRTLRLDSSLKSDTWRQNFRPARLAKLAVRSRYVASLNKTDRASQAPVLRFRPAGQLQSKSKLIDESRPRRLKLGTTLYNSWLAFDSKPIEWATPGTDLYKNAQLNTVLKSALINLNQDYYNDQTPEWGNWWNWELGISRSVNNTLVILYDDLPSTLIDKYNLATRHFVRDPRYLAEGSGAPYSTTKNAFTSTGGNRIDSAMVVFVRGLLANDPGEISAAVTSVPEVLNTVQSGDGFYKDGSFIQHKDLPYSGTYGQVLLNGLGLIKNSVAGTPWDFSVEDNRRIYDVIRQAFLPLLHEGKMPDAVNGRSISRKNGQDQDVGASVMNAIALFVNGAPPEEKRHIEQVLKAQLNTQLDARKMPSYYASAALRYRSRLRIYTHPDRLRYRTAGGKLYADMDRLIYQGTNYLAVVAMHSNRTGSYECINNENANDFLSAWTLRTNPLPNDDQYRDYWPVVDSRFLPGTTSAGEQGWCDEQYRVTQLGRANIAWAGGNTLNKWASASMHLKVPTYSLKAKKSWFVTIALPITMGSQISSSSPHRPIASRSHRSHSAKVLVDGITIASEGLALAINRIRRAIKGNNACSPIAKSHRYDRVRAYIQGNWADIGTSSGKVSAQFLNSRCATASAFDNHYAWVVFPSGSASPSVNADITLLANDAKVQHRRDQSSPSRRVRAIIRSTVGGIHALTPMSLIMTPTTQGYQIAVSSPRRDSRVSFQLPDNAIPFHISSDPDKRVSLNGDIVSVNMTNLRGSSYSFELSKNK |
| Α-factor | MRFPSIFTAVLFAASSALAAPVNTTTEDETAQIPAEAVIGYSDLEGDFDVAVLPFSNSTNNGLLFINTTIASIAAKEEGVSLEKREAEA |
| SUMO | MSDSEVNQEAKPEVKPEVKPETHINLKVSDGSSEIFFKIKKTTPLRRLMEAFAKRQGKEM DSLRFLYDGIRIQADQTPEDLDMEDNDIIEAHREQIGG |


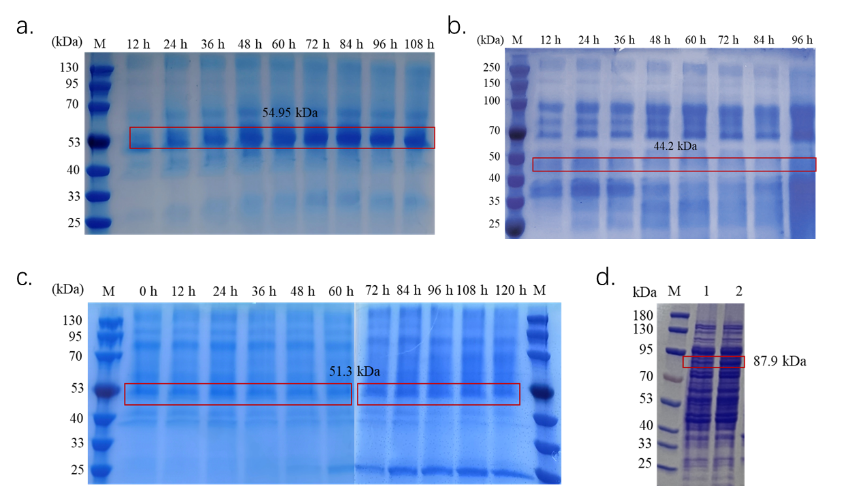


Figure 1 The SDS-PAGE gel electrophoresis image of HHya1, LHya2, SHya3, and EHya4. The expression of HHya1 (a), LHya2 (b), and SHya3 (c) using Pichia pastoris at different time points, and the expression of EHya4 using BL21(DE3) (d).

Figure 2. The pH stability of four hyaluronidases
